# The worms that invade your brain

**DOI:** 10.1192/j.eurpsy.2021.1939

**Published:** 2021-08-13

**Authors:** G. Marinho, J. Peta, S. Vieira, M. Marguilho

**Affiliations:** 1 Clinica 6, CHPL, Lisbon, Portugal; 2 Psychiatry, Centro Hospitalar Psiquiátrico de Lisboa, Lisboa, Portugal; 3 Clínica 5, Centro Hospitalar Psiquiátrico de Lisboa, Lisboa, Portugal

**Keywords:** neurocysticercosis, psychiatric comorbidity, neuropsychiatric manifestations

## Abstract

**Introduction:**

Neurocysticercosis is a parasitic infection of the central nervous system and caused by the pork tapeworm Taenia solium. Humans become infected after consuming undercooked food or water contaminated with tapeworm eggs, or through poor hygiene practices. The clinical manifestations of neurocysticercosis (NCC) largely depend on the number, type, size, localization, and stage of development of cysticerci, as well as on the host immune response against the parasite. Seizures are the most common manifestations of NCC (70–90%) of patients, followed by headache (38%), focal deficits (16%) and signs of intracranial hypertension (ICH) (12%), but psychiatric symptoms can also be seen.

**Objectives:**

Literature review on neuropsychiatric manifestations of neurocysticercosis, based on a clinical case.

**Methods:**

Pubmed search using the keywords neurocysticercosis, psychiatric comorbidity, neuropsychiatric manifestations.

**Results:**

We present a clinical case of a 29-year-old male patient, with history of an epilepsy, that immigrated to Portugal with his family from Cape Verde for specialized medical care. He presented to the ER with an acute psychotic episode characterized by disorientation, persecutory ideation, psychomotor agitation and violent behavior. Brain CT scan showed multiple calcifications in cerebral parenchyma and CSF was positive for antibodies against T. solium.

**Conclusions:**

The polymorphous symptomatology seen in NCC is mimicked only by neuro-tuberculosis and neurosyphilis in developing countries, and multiple sclerosis in the Western countries. Psychiatric symptoms are a part of the clinical presentation of infectious diseases. It is important to consider NCC in endemic areas presenting with psychiatric symptoms, especially those showing poor response to the standard treatment and in those with history of seizures.

**Disclosure:**

No significant relationships.

